# Dental DNA as an Indicator of Post-Mortem Interval (PMI): A Pilot Research

**DOI:** 10.3390/ijms232112896

**Published:** 2022-10-25

**Authors:** Ilenia Bianchi, Simone Grassi, Francesca Castiglione, Caterina Bartoli, Bianca De Saint Pierre, Martina Focardi, Antonio Oliva, Vilma Pinchi

**Affiliations:** 1 Forensic Medical Sciences, Department of Health Science, University of Florence, Largo Brambilla 3, 50134 Florence, Italy; 2 Department of Law, University of Macerata, Via Crescimbeni, 30/32, 62100 Macerata, Italy; 3 Section of Anatomic Pathology, Department of Health Sciences, University of Florence, 50134 Florence, Italy; 4 Section of Legal Medicine, Department of Health Surveillance and Bioethics, Catholic University of Sacred Heart, Fondazione Policlinico Universitario A. Gemelli IRCCS, Largo Francesco Vito, 1, 00168 Rome, Italy

**Keywords:** PMI, ADD, NGS, forensic odontology, teeth

## Abstract

Teeth have proven to be a reliable source of DNA for forensic analysis as the pulp is rich in cells and protected from damaging factors and contamination by dental hard tissues. The pilot study aims to evaluate the feasibility of Next-Generation sequencing analysis on dental pulp to detect genetic mutations in DNA caused by post-mortem cell necrosis. We used a 56-gene oncopanel kit on a sample of 17 teeth extracted from living patients. Time of the tooth avulsion was assumed as death of the individual and Post-mortem Interval (PMI) was the time elapse since the DNA extraction and analysis. Days and Accumulated Degree Days (ADD) were assumed as measures of PMI that ranged between 0 to 34 days. Only 38 of the 56 considered genes proved to be affected by mutations (101), thus being of forensic interest. More specifically, 14 mutations occurred only in a specific range of PMIs/ADD; 67 were detected (alone or as clusters of the same gene) at specific PMI/ADD; 22 occurred at every PMI/ADD, except for some specific intervals. Since dental pulp was not targeted by any oncological diseases and all teeth were intact, vital, and from patients with unremarkable medical history, it could be assumed that mutations were due to post-mortem DNA changes induced by pulp death and the increasing time elapse since death. This pilot study found encouraging results in the application of NGS analysis on dental DNA, especially for PMIs of several days for which the traditional tools for PMI estimation have limitations. Further research on a larger sample of PMI and validation research on a larger sample of PMI and validation of the results are indeed necessary.

## 1. Introduction

A reliable estimation of the post-mortem interval (PMI) is a pivotal step in criminal investigations, since the criminal system requires that every fact is proven beyond any reasonable doubt [1]. Despite this, a practical and reliable tool for this task has not been reported yet and the most commonly used algorithm remains the combination of different parameters (in particular, the body and environmental temperatures) through the so-called “compound method” [2,3].

Tools based on physical and/or chemical parameters can be biased because they are often developed considering “ideal”/experimental conditions and thus, especially when the crime scene is in an open environment, their estimates can be influenced by several confounding variables [4,5,6,7,8]. Another limitation is given by the fact that most of these tools are valid only for estimates of the early (<72 h) PMI, while for longer PMIs the measurement errors are generally so relevant that the estimated value is of scarce inductive and legal significance [3,9,10]. Currently, alternative methods are mainly focused on post-mortem biochemical or histological changes [11,12,13,14,15,16,17,18] and on the noninvasive observation of structural alterations (such as the decrease in corneal thickness) [19]. 

In general, several genetic tools for PMI estimation have been developed in the last few years. Indeed, post-mortem genetic testing may be used for this task through the analysis of the degradation products of nucleic acids, which tend to progressively increase after death [20,21,22,23,24,25]. These techniques have been reported to allow PMI estimation, even after more than 36 h since death [23,26]. For instance, Rubio et al. found that if the biological samples are properly stored (in particular, at an adequate temperature), post-mortem quantitative analysis on nucleic acids products can be adequately performed up to (at least) 18 months [27]. A significant limitation of post-mortem genetic testing as a tool for PMI estimation is given by the relatively high susceptibility of nucleic acids to external factors, such as germs, moisture, heat and UV light, which can alter and damage their structures [23]. 

One of the least exposed DNA sources is represented by the dental pulp, which is protected by the hard tissues of the tooth: indeed, nucleic acids of the pulp of human teeth are reportedly more stable and less sensitive to temperature variation than many other tissues of the organism [28,29,30].

One of the main issues in applying DNA in PMI estimation is the lasting stability of this nucleic acid over time, thus the slightest post-mortem (PM) variations are less detectable through normal genetic analysis techniques. Nevertheless, some studies reported the integrity of the DNA chain as a useful substrate only in the estimation of some ranges of PMI. More specifically, concentration and integrity decrease significantly within 1 month after death, then stabilize between 1 and 12 months, then degradation restarts at 18 months after death [23,27,31,32].

In this study, we performed a high-throughput sequencing (Next-Generation sequencing—NGS) on the pulp of 17 teeth, using a 56 gene panel to evaluate the feasibility of analyzing dental pulp DNA to estimate the PMI. In agreement with the previous study conducted by Ferreira PG et al. (2018) [33], some genes reactivate and/or hyper-activate their post-mortem transcription by modulating mRNA production in relation to the time since death.

The hypothesis of pilot study is that the post-mortem genetic activity, induced by cell necrosis in the pulp of extracted teeth, can be used as a measure of PMI. Furthermore, we investigated if the genetic post-mortem activity can be detected by oncopanels normally used for NGS analysis in clinical cases, which makes it possible to reveal mutations induced by tumor activity, including cell necrosis. After all, no previous research has been conducted on a specific genetic panel to detect postmortem DNA variations, thus large oncopanels could be deemed possibly useful for such an analysis.

## 2. Results

A total of 17 teeth extracted from 17 patients (5 males vs. 12 females) were initially eligible for this study. The PMI varied from 0 to 34 days (median: 11.00; mean: 14.50; standard deviation: 10.50), while the ADD varied from 0 to 798.80 (median: 265.98; mean: 326.48; standard deviation: 237.54) (Table 1).

We found 328 mutations in 47 genes, with 125 mutations (in 38 genes) having a prevalence > 5%.

Overall, 101 mutations were considered of interest for the purposes of the study, occurring in an irregular fashion that allowed attribution to specific PMI/ADD or specific ranges of PMIs/ADD. In particular, Table 2 shows the mutations that were found only during specific time intervals.

Moreover, Table 3 reports the mutations found only at specific PMIs.

Finally, Table 4 shows the mutations that were not found only for specific PMIs.

## 3. Discussion

Teeth can be considered a promising source of DNA for forensic purposes since the risk of low template DNA is lower because the pulp has high cellularity (there are about 11,000 odontoblasts and 1000 fibroblasts per mm^2^) and dental hard tissues (enamel and dentine) protect nucleic acids from damaging factors such as heat, germs, UV light and moisture [34,35,36,37]. Indeed, dental DNA concentration remains relatively high in the first 1–2 days post-mortem (dropping after 10 days) even in burnt corpses [38]. However, the analysis of dental DNA raises several issues. Firstly, it can be technically complex to obtain DNA from teeth due to the surrounding hard tissues; secondly, the quantity of dental DNA can be affected by physiological factors, as individual ageing, or tooth type, or dental pathologies such as caries or treatments; lastly, the enamel contains many PCR inhibitors, such as calcium [34]. 

We performed targeted NGS on 17 sound teeth extracted from healthy patients. The DNA was extracted from the pulp of each tooth at a set ADD/PMI. The term PMI is used since the tooth extraction interrupts the blood perfusion of the dental pulp, thus simulating the death of the patient. We chose teeth of clinical cases, rather than those of forensic cases, in order to have certain PMIs, whilst in a forensic context the time of death must be generally estimated. Both PMI and ADD were considered because, in non-experimental conditions, temperature variations analysis enable a reduction in the error in estimating the time since death [5,39].

The fact that 16/17 considered teeth were molars should be regarded as a relevant factor for studies on post-mortem DNA, because in forensic cases molars have been associated with the highest DNA yield [34]. Moreover, molars (rather than incisors or canines) should be preferred candidates for forensic analysis because of their position in the dental arches, which protects them (and then their DNA) from traumas and heat. Extracting molars should be preferred also to minimize the demolition of the anterior part of the smile, which is important for the identification of unknown bodies. Moreover, extracting posterior teeth rather than incisors and canines limits the invasiveness of the procedure, a criterion that is often considered by public authorities that request/authorize the forensic investigation. 

According to the definition of somatic mutations given by Karki et al. provided for DNA variants [40], the NGS found 328 *somatic mutations* in 47 out of the 56 genes of the used panel. We chose this specific panel because it has been validated for detecting somatic mutations due to oncological diseases affecting different tissues which discard the multiple polymorphisms [41,42,43]. Since dental pulp is not targeted by any oncological diseases and all teeth were intact, healthy, with vital pulp and came from patients with an unremarkable medical history, the detected mutations could be solely attributed to post-mortem DNA changes. Previous research proved the occurrence of an active and continuous regulation of mRNA transcription even after cell death by identifying genes that were differentially expressed between ante-mortem and post-mortem blood samples at different PMI intervals [33].

Finally, mutation variants with an allelic frequency <5% were excluded from our study, an additional safety margin to minimize intrinsic analysis error. Therefore, 125 mutations (belonging to 38 genes) were investigated.

The absence of significant mutations (Table 2 and Table 3) at T0 (time of death) was expected since the chosen panel is normally used for testing the occurrence of neoplastic pathologies, thus the target genes are not mutated under physiological conditions [44], which featured these healthy dental pulp samples. 

In total, 27 of 128 mutations were of scarce interest because they did not occur in a fashion that allows an association with specific PMI/ADD. For instance, two mutations (c.*35C>T, and c.*36A>C) of *CSF1R* were found at every PMI/ADD, with prevalence values ranging from 50 to 100%. The remaining 101 variants were considered of interest for the purposes of the study, occurring in an irregular fashion that allowed attribution to specific PMI/ADD or specific ranges of PMIs/ADD. In detail, 14 mutations occurred only in a specific range of PMIs/ADD (Table 2). For instance, *c.740A>G* variant of *ABL1*, *c.451-9G>A* variant of *KRAS* and *c.1092-41G>A* variant of *SMARCB1* only occurred at PMI 14-15 but were found neither before nor after this range. We also found 67 mutations that only occur (alone or as clusters of mutations of the same gene) at specific PMI/ADD (Table 3). For instance, at every PMI/ADD there is a variant of *KDR*, but the combination of four variants (*c.2615-37dupC*, *c.1416A>T*, *c.2615-50_2615-40del* and *c.2615-34_2615-33insT*) is specific for PMI 5 after the death. Finally, some mutations occur at every PMI/ADD but for some specific intervals: for instance, *ERBB2* is always mutated but for PMI 14 and 15 (Table 4). 

In our opinion, the preliminary results of this study should be considered for further validation in larger cohorts of teeth, since the combination of the three kinds of mutation detected here could be of potential interest for the estimation of PMI. Hence, these findings cover the first month (34 days) as a relatively long period after death, for which the PMI estimation could result challenging on the basis of the traditional and validated methods. Consistently with previous evidence, our findings indicate that dental DNA analysis should be mainly considered for the estimation of the late PMI [23], since the overall number of mutations increases as the PMI increases.

An additional consideration is that data obtained through the current pilot study do not allow inference on the causes of our findings. Therefore, these results should be interpreted as supporting the feasibility of this technique for the PMI investigation, albeit a larger number of teeth must be evaluated to perform statistical analysis and thus to validate the procedure. Since the mutations of interest occur at specific PMI with relatively high prevalence values, the risk of false positives is relatively low, and our findings can be deemed unlikely due to chance. At the same time, the mutations, that were not detected only at specific PMIs, showed relatively high prevalence in the other PMI intervals, suggesting that it is unlikely that these findings are false negatives.

## 4. Materials and Methods

### 4.1. Sample Collection and Storage

The study considered human teeth extracted for clinical reasons in living patients, rather than teeth of cadavers of forensic cases, for which the time of death is often unknown and PMI must be actually estimated. Teeth were extracted for clinical reasons from patients at the Dental Department of Careggi University Hospital in Florence, Italy, and the study obtained approval from the local Ethical Committee (n. 15208/2020). Consent for research and publication was obtained in all the cases and data were processed in compliance with European Union law (GDPR).

Inclusion criteria were:

- Unremarkable medical history of patients.
- Intact teeth, sound and unrestored.
- Normal response to pulp vitality tests. Thermal tests were performed before extractions to exclude pathologies or necrosis of dental pulp.
- Permanent and completely mineralized premolars, molars, and canines of both dental arches. These teeth were chosen to yield the higher quantity of DNA and to minimize the demolition of anterior parts the mouth of the cadaver.

Exclusion criteria were:

- Incisors.
- Decayed, fractured, or damaged teeth.
- Treated teeth with conservative, endodontic, or prosthetic therapies.
- Non vital teeth or abnormal results of vital pulpal tests.
- Medical history positive for relevant diseases (e.g., cancer, diabetes) or chronic drug treatments.

An eligible sample composed of 17 teeth extracted from 17 different patients was then selected for the study. Sex, age, tooth position, and date of the avulsion were registered for each patient.

The tooth avulsion was assumed as time of the death and the PMI was the time elapse between the avulsion and the DNA extraction and analysis.

Immediately after the avulsion, teeth were rinsed with a physiological solution and placed inside a sterilization sachet, on which the identification code of the tooth itself was reported, stored inside a closed plastic container. Samples were stored at not standardized temperatures and conditions to simulate real corpses conservation and both PMI and ADD (accumulated degree-days) were registered. All the samples were progressively inserted in the plastic container that contained a datalogger (IButton DS1923 Hygrochron Temperature/Humidity Data Logger, iButtonLink Technology, LLC, Whitewater, WI, USA), which is a device programmed to record, at intervals of 15 min, the temperature and humidity inside the box placed at room temperature. The data logger remained active throughout the period of the extraction phase, gradually reprogrammed according to its recording autonomy, using the appropriate Software (Express-Thermo, Eclo Solutions, 2014 version, Leiria, Portugal). PMI was calculated as the time since the avulsion of the tooth (entailing the interruption of the blood supply). Instead, ADDs were calculated as: (T maximum + T minimum)/2 − T threshold, where T maximum indicates maximum temperature reached during the day, T minimum, the minimum temperature reached during the day, and T threshold, which represents the value above which the phenomenon examined takes place; 0 °C was considered here as freezing inhibits nucleic acid degradation [45]. 

### 4.2. Dental Pulp Extraction

The extraction of pulp tissue from each tooth was performed at a set ADD (each extraction at a different ADD). When the tooth reached the set ADD, it was frozen at −20 °C in order to block the degradation of nucleic acids. An operator extracted pulp tissues through a coronal-apical groove on the midline of the buccal surface of the frozen teeth in a sterile environment at the Section of Forensic Sciences of the Health Sciences Department of the University of Florence, Italy. 

The same procedure was performed for each tooth by operators wearing individual protective devices replaced for each sample and using only sterile instruments and careful disinfection of the surfaces between each sample to avoid possible contamination of the DNA. 

To perform pulp tissue extraction, the following procedure was adopted: the sulcus had a depth of about 1 mm at the apical level and 2 mm at the coronal level, measured by a periodontal probe in such a way as to retain the enamel/cement and superficial dentin, and was performed with a finishing bur, mounted on a turbine, with abundant irrigation. Then, the tooth was wrapped inside a transparent film, in order to prevent the dispersion of any fragments during the break, and it was inserted in a vice that held it in position; a hammer and a thin lever were used to open the tooth in correspondence with the previously created guide sulcus; the pulp tissue was removed using tools such as tweezers, endodontic files, small excavator. The pulp fragments were deposited in the appropriate histological cassettes, on each of which the identification code of the sample and its PMI were reported. The cassettes were inserted, two at a time, into the 60 mL Securbiop biopsy container (SecurBiop^®^ e FORMALeasy^®^, Techingreen Srl, Teramo, Italy) with 4% buffered formaldehyde up to 12 h at low temperature to fix pulp specimens according to the DNA extraction and analysis procedures described by Berrino et al. [46,47] applied in the next step and to the fixed-DNA preservation procedures. 

### 4.3. DNA Extraction and NGS Analysis

The Genomic DNA (gDNA) was extracted using “MagCore genomic DNA FFPE one-step” (Diatechlabline pharmacogenetics, Jesi, Italy) for the pulp fragments previously fixed in formalin. Since no previous studies experimented specific genetic panels for analysis of post-mortal DNA degradation, we hypothesized that genetic alterations induced by post-mortal cell necrosis could be similar to those induced by neoplastic phenomena, thereby being possibly detectable by oncopanels. Using the “Myriapod NGS-L T 56G Oncopanel Kit” (Diatechlabline pharmacogenetics, Jesi, Italy), the quantity and quality (fragmentation) of the gDNA were evaluated through quantitative polymerase chain reaction (q-PCR, Rotor-gene, Qiagen, Hilden, Germany). Subsequently, the extract was amplified through multiplex PCR. After the purification and indexing step with the “barcodes”, the libraries obtained, after enrichment, were quantified (minimum concentration equal to 2 nanomoles) with Qubit dsDNA HS Assay kit (Invitrogen by Thermo Fisher Scientific, Midland, Canada). Myriapod Oncopanel (Diatechlabline pharmacogenetics, Jesi, Italy), which targets over 500-point mutations in 56 genes, was considered as the panel. Emulsion PCR was performed using the Ion One Touch 2 instrument (Life Technologies, Grand Island, The Netherlands). Using Ion One Touch ES Ion Torrent (Life Technologies, Grand Island, The Netherlands), Dynabeats MyOne Streptavidin C1 Beads (Invitrogen by Thermo Fisher Scientific, Midland, ON, Canada) were added to the pool. Sequencing was performed with the Ion S5 System Ion Torrent platform (Invitrogen by Thermo Fisher Scientific, Midland, Canada). The analysis of the sequencing results and the identification of any genetic variants in the target regions (the “variant-calling” after “target sequencing”) was carried out using the “Myriapod NGS Data Analysis Software” (Myriapod NGS Analysis software e Myriapod workstation NGS, Diatech Pharmacogenetics srl, Jesi, Italy). The reference genome for the sequences obtained was GRCh37. Only the sequences that had a quality index (PHRED score) of at least Q > or = a 20 were considered, while the minimum coverage considered in each sample was 100 reads per amplicon. The non-synonymous mutations were selected using on-line genetic databases (such as COSMIC, LOVD, PUBMED, Clinvar, last access on 10 May 2022) for the variants already described, while for the genetic polymorphisms (Single nucleotide Polymorphism, SNP), dbSNP and 1000 Genome. For the mutations not yet described, the pathogenetic prediction “tools” of the variant were used, such as WebAnnover and Provean (SIFT and Pholyfen).

## 5. Limitations

Since we considered teeth extracted from clinical patients, we could not collect more than one tooth per patient. Therefore, different PMIs/ADD corresponded to different cases and not enough teeth were collected to cover the entire 34-day period.

The small pilot sample does not allow a correlation between the variants and each PMIs, as well as the inference for mechanism behind mutations such as transversions, transitions, deletions, etc. 

## 6. Conclusions

This study confirmed that dental DNA, studied through NGS, can represent a promising source of data for the estimation of late PMI for forensic purposes. Out of the 56 investigated genes, only 39 proved to be of some interest, showing mutations at certain PMIs or in specific PMI intervals. The absence of mutations at T0 (time of death) and the increase in occurrence of mutations at late PMIs, indicate that mutations were due to post-mortem DNA variations. 

This pilot study represents a novel research line aimed at identifying new methods for PMI estimation. The results showed the feasibility of NGS on post-mortem samples, but the occurrence of mutations even during and after cell necrosis cannot be explained without further research, since there are no previous studies that have investigated this specific phenomenon.

Future studies can use customized panels limited to the genes that resulted here of interest for PMI estimations, thus entailing a cost reduction.

The study is affected by limitations given the small number of DNA samples and PMIs considered for the research, apart from the limitations due to the fact that only one DNA extraction was performed for each tooth/individual.

A prosecution of the research is ongoing on a larger number of teeth from both clinical and forensic cases, which is designed to grant an even coverage of the first month after death. Secondly, future research will address the validation of the technique on multiple samples from the same individual and on forensic cases, thus possibly enabling inference about mechanisms behind PM mutations.

## Figures and Tables

**Table 1:** Data set of eligible samples. Tooth position, PMI and ADD.

CASE	TOOTH *	PMI (Days)	ADD
1	ULTM	T0 (0)	0
2	ULTM	1	23.84
3	LRTM	5	92.9
4	LRTM	6	114.73
5	URTM	7	134.56
6	LRTM	8	183.18
7	ULC	9	216.03
8	LRTM	10	240.88
9	URTM	11	265.97
10	LRTM	14	349.75
11	ULTM	15	372.09
12	URTM	19	402.55
13	ULTM	22	531.78
14	LLTM	26	549.86
15	LRTM	27	570.19
16	LRTM	32	702.96
17	ULTM	34	798.80

* LRTM: lower right third molar; LLTM: lower left third molar; URTM: upper right third molar; ULTM: upper left third molar; ULC: upper left canine.

**Table 2:** Mutations found only during specific time intervals.

PMI (Days)		0	1	5	6	7	8	9	10	11	14	15	19	22	26	27	32	34
ADD (°C)		0	23.84	92.90	114.73	134.56	183.18	216.03	240.88	265.97	349.75	372.09	402.55	531.78	549.86	570.19	702.96	798.80
GENES	MUTATIONS																	
ABL1	c.740A>G										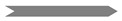							
APC	c.4785C>A							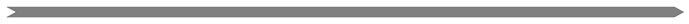										
ATM	c.1229T>C		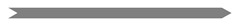															
	c.8671+49A>T								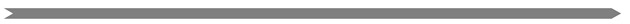									
	c.5177+17A>G								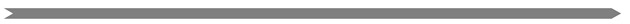									
FLT3	c.2053+23A>G												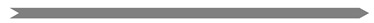					
HRAS	c.-10C>T							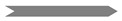										
JAK3	c.381T>G							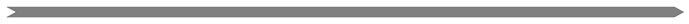										
	c.2164G>A									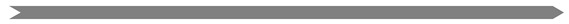								
KRAS	c.451-9G>A										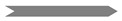							
	c.450+31T>C								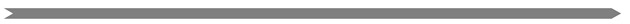									
PDGFRA	c.2472C>T										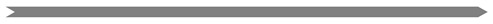							
PIK3CA	c.1173A>G									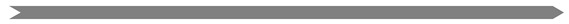								
SMARCB1	c.1092-41G>A										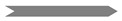							

Dark-grey cells represent PMIs in which mutations were found.

**Table 3:** Mutations that were found only during specific PMIs.

PMI (Days)		0	1	5	6	7	8	9	10	11	14	15	19	22	26	27	32	34
ADD (°C)		0	23.84	92.90	114.73	134.56	183.18	216.03	240.88	265.97	349.75	372.09	402.55	531.78	549.86	570.19	702.96	798.80
GENES	MUTATIONS																	
APC	*c.4479G*>*A*																	
	*c.4785C*>*A*																	
	*c.4373C*>*A*																	
	*c.2662G*>*T*																	
ATM	*c.8850+35delT*																	
	*c.8671+49A*>*T*																	
	*c.5177+17A*>*G*																	
	*c.5131A*>*G*																	
	*c.1010G*>*A*																	
	*c.8988-9delT*																	
CDKN2A	*c.222C*>*T*																	
CSF1R	c.*24C>*T*																	
EGFR	*c.2429G*>*C*																	
	*c.2514G*>*T*																	
	*c.2470-7G*>*T*																	
ERBB2	*c.2088G*>*T*																	
	*c.2307+42C*>*T*																	
ERBB4	*c.977G*>*T*																	
FGFR2	*c.1650T*>*A*																	
	*c.1622T*>*G*																	
FLT3	*c.1992G*>*C*																	
IDH1	*c.315C*>*T*																	
KDR	c.*27T>*C*																	
	*c.2615-37dupC*																	
	*c.1416A*>*T*																	
	*c.2615-50_2615-40del*																	
	*c.2615-34_2615-33insT*																	
	*c.889G*>*A*																	
	*c.1444T*>*C*																	
	*c.2615-42_2615-40del*																	
KIT	*c.2394C*>*T*																	
MAP2K1	*c.711G*>*A*																	
	*c.1069-3C*>*A*																	
	*c.1141C*>*T*																	
MET	*c.487_488delTC*																	
NOTCH1	*c.4779G*>*T*																	
NPM1	*c.847-6_847-5delTT*																	
	*c.849T*>*C*																	
NRAS	*c.291-38G*>*T*																	
	*c.450+18G*>*A*																	
PDGFRA	*c.2002+5G*>*A*																	
PTEN	*c.848C*>*A*																	
	*c.802-34C*>*T*																	
	*c.-1C*>*A*																	
	*c.253+2T*>*G*																	
	*c.1074G*>*A*																	
	*c.926C*>*T*																	
	*c.509G*>*A*																	
RB1	*c.462G*>*A*																	
RET	*c.1982A*>*G*																	
	*c.2712C*>*G*																	
SMAD4	*c.728G*>*A*																	
	*c.688G*>*A*																	
SMARCB1	*c.1092-62G*>*A*																	
	*c.177G*>*A*																	
SMO	*c.1575G*>*A*																	
	*c.1605_1625del*																	
STK11	*c.597+14A*>*G*																	
TP53	*c.503A*>*C*																	
	*c.753C*>*G*																	
	*c.455C*>*T*																	
	*c.74+1G*>*T*																	
TSC1	*c.437C*>*T*																	
	*c.2742+6C*>*A*																	
	*c.2742+23C*>*T*																	
VHL	*c.464-50T*>*G*																	
	*c.321C*>*G*																	

Dark-grey cells represent PMIs in which mutations were found.

**Table 4:** Mutations that were not found only for specific PMIs.

PMI (Days)		0	1	5	6	7	8	9	10	11	14	15	19	22	26	27	32	34
ADD (°C)		0	23.84	92.90	114.73	134.56	183.18	216.03	240.88	265.97	349.75	372.09	402.55	531.78	549.86	570.19	702.96	798.80
GENES	MUTATIONS																	
APC	*c.4479G*>*A*																	
EGFR	*c.2361G*>*A*																	
	*c.1839C*>*T*																	
	*c.2429G*>*C*																	
	*c.2514G*>*T*																	
	*c.2470-7G*>*T*																	
ERBB2	*c.2086-29G*>*A*																	
	*c.2088G*>*T*																	
	*c.2307+42C*>*T*																	
	*c.922G*>*T*																	
	*c.1963A*>*G*																	
	*c.2088G*>*T*																	
	*c.2308-12C*>*A*																	
FGFR3	*c.1953G*>*A*																	
	*c.843A*>*C*																	
	*c.840C*>*T*																	
FLT3	*c.1310-3T*>*C*																	
PTEN	*c.802-29C*>*T*																	
	*c.848C*>*A*																	
	*c.802-18C*>*T*																	
	*c.802-23C*>*T*																	
	*c.802-3dupT*																	

Dark-grey dots represent the only PMIs in which mutations were not found.

## Data Availability

Data are available on reasonable request to the corresponding author.
